# Association between treatment for gonorrhoea and chlamydia and lower condom use in a cross-sectional study of female sex workers in southern India

**DOI:** 10.1136/bmjopen-2015-009774

**Published:** 2016-05-18

**Authors:** Marianne Legendre-Dugal, Janet Bradley, Subramanian Potty Rajaram, Catherine M Lowndes, Banadakoppa M Ramesh, Reynold Washington, Stephen Moses, James Blanchard, Michel Alary

**Affiliations:** 1 Centre de recherche du CHU de Québec, Québec, Canada; 2 Département de médecine sociale et préventive, Université Laval, Québec, Canada; 3 CHARME-Project, Bangalore, Karnataka, India; 4 Karnataka Health Promotion Trust, Bangalore, Karnataka, India; 5 Public Health England, London, UK; 6 St. John's Research Institute, Bangalore, Karnataka, India; 7 Department of Community Health Sciences, University of Manitoba, Winnipeg, Manitoba, Canada; 8 Centre for Global Public Health, University of Manitoba, Winnipeg, Manitoba, Canada; 9 Institut national de santé publique du Québec, Québec, Canada

**Keywords:** SEXUAL MEDICINE

## Abstract

**Objectives:**

To assess whether having received grey packets containing treatment for gonorrhoea and chlamydia was associated with condom use among female sex workers (FSWs) in 5 districts of southern India covered by the *Avahan* programme where both periodic presumptive treatment (PPT) and syndromic management were used to control these sexually transmitted infections (STIs) among FSWs.

**Setting:**

Cross-sectional study of FSWs recruited in the field in 5 districts of southern India (Bangalore, Belgaum, Bellary, Guntur and Mumbai) in 2006–2007.

**Participants:**

1378 self-identified FSWs out of 1442 were approached to participate in the study (participation rate: 95.6%). The only exclusion criterion was to be aged <18 years.

**Primary and secondary outcome measures:**

Consistent condom use (CCU) with new or occasional clients, and with the most recent repeat client as assessed using a questionnaire administered through face-to-face interviews.

**Results:**

Using the Poisson regression to model the association between the number of grey packets received in the past 3–12 months and reported CCU, adjusting for factors associated with condom use and other potential confounders in our data, CCU was lowest among FSWs who had received ≥3 grey packets in the past 3–12 months with their new or occasional clients (adjusted prevalence ratio (APR): 0.70, 95% CI 0.57 to 0.84, p<0.001) and with the most recent repeat client (APR 0.63, 95% CI 0.51 to 0.78, p<0.001). Tests for trends showed that CCU with both types of clients decreased with the number of grey packets received (p<0.001).

**Conclusions:**

Since we could not distinguish grey packets used for PPT from those given for syndromic management, these results could be either due to a perception of protection conferred by PPT or by the fact that inconsistent condom users are more at risk for STIs. Further research on the potential disinhibiting effect of PPT is warranted.

**Strengths and limitations of this study**

- This study is part of the evaluation of *Avahan*, an HIV prevention programme targeted at high-risk groups, notably female sex workers, in India.
- In this study, the use of treatment for gonococcal and chlamydial infection, in the context of periodic presumptive treatment and syndromic management of female sex workers, was associated with lower consistent condom use.
- The strengths of this study include its large sample size across five Indian districts and the fact that the hypothesis that inappropriate counselling in clinical settings may lead to a decrease in condom use is based on the results of a previous qualitative study conducted in the same population.
- The main limitation is that it was impossible to determine if the treatment was given presumptively or in the context of syndromic management.
- This association that we observed could thus be due to a perception of protection conferred by the treatment or because inconsistent condom users are more at risk for sexually transmitted infections.

## Background

Sexually transmitted infections (STIs) are considered to increase the risk of HIV transmission, since both infectiousness among HIV-infected people and susceptibility for HIV acquisition among those uninfected are increased by STIs. There has thus been interest in treating STIs as a way to prevent HIV acquisition.^1–4^ In resource-limited settings, where sophisticated laboratories are not regularly accessible, STIs are often treated presumptively or on the basis of symptom-based algorithms. Unfortunately, among women, the sensitivity and specificity of syndromic case management tend to be very low when the vaginal discharge syndrome is used as an indication for treating gonorrhoea and chlamydia, with the latter infections sometimes not even associated with symptoms of vaginal discharge.^5^ Moreover, gonorrhoea and chlamydia are often characterised by an absence of symptoms, especially among women.^5^ ^6^ In settings where STI prevalence is high and concentrated in certain core groups, it is assumed that periodic presumptive treatment (PPT) can be effective as an HIV prevention strategy.^3^ ^7–9^ However, this strategy has to be part of a more complete programme to have long-term sustainability. The programme has to include communication for behavioural change with regard to risky sexual behaviour, in particular condom use, as well as structural components and clinical services other than PPT.^10^ ^11^

*Avahan*, the India AIDS initiative of the Bill & Melinda Gates Foundation implemented since 2003, is a large-scale HIV prevention programme whose main objective was to reduce HIV prevalence among high-risk groups such as female sex workers (FSWs), men who have sex with men (MSM), people who inject drugs (PWID) and clients of FSWs in the six states of India with the highest HIV prevalence.^12^ ^13^ The intervention, which has been described in more detail elsewhere,^12^ includes behavioural change communication with the promotion of condom use using peer educators, free distribution of commodities (condoms, clean syringes for PWID), community development, interventions against stigma and discrimination, and STI care. The latter used an ‘essential service package’ for FSWs that was offered exclusively at *Avahan* FSW-dedicated STI clinics and included PPT for gonorrhoea and chlamydia (recommended quarterly), syndromic case management of women coming to the clinic with STI symptoms and serological testing, followed by treatment, when appropriate, for syphilis twice a year. The target of the strategy was to attain one clinic visit per FSW every 3 months.^14^ Anecdotally, programme implementers observed that in their communication with patients, physicians were not always very clear about the STIs for which the treatment was protective and did not give enough adequate information adapted to FSWs' understanding, which could have led some women to think that the treatment given may protect directly against HIV or other STIs than those for which treatment was given. Such beliefs were also observed in a qualitative study carried out among FSWs in Guntur district, state of Andhra Pradesh.^15^ Although a few previous studies did not show a decrease in condom use following the implementation of PPT among FSWs,^6^ ^8^ data on this issue remain sparse and a concern subsists about a disinhibiting effect of STI treatment in relation to condom use. In an order to constantly improve prevention programmes, the objective of this secondary data analysis was to determine if an association could be found between the prescription of STI treatment and condom use and, more specifically, if the disinhibiting hypothesis could be plausible in the context of FSWs with their clients in five districts of south India.

## Methods

Under the CHARME-India project (led by the Centre hospitalier *affilié* universitaire de Québec in partnership with Canadian, British and Indian institutions) whose aim was to evaluate the impact of the *Avahan* programme, we conducted detailed behavioural cross-sectional studies among FSWs in five districts of south India between February 2006 and December 2007. Of the 1442 FSWs invited, 1378 (95.6%) participated in the study. Three of the study districts were in the state of Karnataka (Bangalore (n=369), Belgaum and (n=208) Bellary (n=198)), whereas the two other districts were Guntur in the state of Andhra Pradesh (n=208) and Mumbai in the state of Maharashtra (n=395). The interviews were conducted face to face by trained interviewers.

Two sampling procedures were used for the selection of clusters. For fixed sex work sites, such as home-based, brothel-based and lodge-based sites, a conventional cluster sampling was used. For street-based and other public place-based sex work sites, time-location cluster (TLC) sampling was used. Normalised weights were calculated for the complex sampling design. Within selected clusters, the respondents were selected randomly. Districtwide mapping of the sites where FSWs could be found was used as well as information about hours of operation for the TLC sampling. We also approximated the possible number of respondents at different times of the day and of the week. Site maps were then used by the research teams for the sampling frame development. Sampling methods were the same as those reported by Saidel *et al*.^16^

Data from face-to-face interviews with FSWs were collected about their sexual activity with two types of commercial partners: new or occasional clients and the most recent repeat client. The two categories of partners were not mutually exclusive, since every FSW having a most recent repeat client also had new or occasional clients, but not necessarily the opposite.

### Ethical considerations

Participation in the study was on a voluntary basis. The interviews were conducted anonymously, with no names or personal identifiers recorded on the questionnaire. Witnessed verbal consent was obtained from each participant prior to the administration of the questionnaire. This means that a witness independent to the study confirmed in writing that the participant had provided verbal consent. This procedure was preferred to written consent to ensure FSWs anonymity as sex work is illegal in India.

### Dependent variable

The dependent variable, consistent condom use (CCU), with new or occasional clients (clients who are not well known by the FSW and who visited her only once or at most a few times) and with the most recent repeat client (clients with whom FSWs are familiar), was obtained through the survey question: ‘In general, how often is a condom used when you have sex with “type of partner”?’. Interviewers were instructed to explain the meaning of ‘in general’ as the current sex work practice of the participants. There was, however, no time frame specified. Possible answers to this question were: never (0%), sometimes (<50%), frequently (≥50%) or always (100%). We considered FSWs were using condoms consistently with their partners if they answered ‘always’ and inconsistently if they answered ‘never’, ‘sometimes’ or ‘frequently’, in order to create a dichotomous variable.

### Independent variables

To determine the relationship between condom use and treatment for STIs, we assessed whether the variable ‘number of grey packets received in the past 3–12 months’ was associated with CCU among FSWs with their new or occasional clients and with their most recent repeat client, while controlling for variables affecting condom use. The grey packets were used for presumptive treatment of asymptomatic chlamydial and gonococcal infections and contained a single dose of cefixime 400 mg and azithromycin 1000 mg. In the case of syndromic management, the grey packets were mostly used in combination with the green packet that was designed to treat vaginitis agents. Since the grey packets contained a single dose treatment, they were taken on site, in front of the healthcare worker, thus ensuring that the treatment was actually taken. Unfortunately, the questionnaire used in the interviews did not address the issue on the reason why the grey packets were taken (PPT or because of vaginal discharge). An analysis of the data of the *Avahan* computerised information system for the years 2006 and 2007 for the three Karnataka districts (data were not available for Mumbai and Guntur) showed that among the 25 530 grey packets administered to FSWs, 32.7% were used for PPT (*Avahan* programme, unpublished data).

The time frame used (3–12 months) was wide because of interdistrict variations in the start dates of the implementation of the STI essential package for FSWs. In all districts except Bangalore, the programme had started 12 months or more prior to data collection. In Bangalore, it had only started 3 months before the interviews were conducted. The treatment variable was divided into four categories, which were zero, one, two, or three or more grey packets.

On the basis of the literature, we determined a set of sociodemographic, sex work-related and intervention exposure factors that may affect condom use.^17–22^ Each model was adjusted for those variables. Sociodemographic factors included district, age, marital status (currently married, divorced/separated/widowed, never married or devadasi, a particular form of sex work where women are dedicated to gods and goddesses through marriage, who practise traditional, caste-based sex work^23^ ^24^), literacy and age at first sex. Sex work-related factors included age at first sex work, main place of solicitation of clients (brothel, home, public places or rented room/lodge or other), main place of entertaining (with the same categories as main place of solicitation), having sex work as sole income, if the FSW is usually under influence of alcohol when having sex with a client, if the partner is usually under influence of alcohol when having sex with the FSW and if the FSW solicits her clients independently or through a middleman or a pimp. Intervention exposure variables, other than the number of grey packets received, included the number of times of being contacted by intervention staff in the past month, the number of condom demonstrations seen in the past month and the time since first contacted by intervention staff.

### Statistical analysis

Statistical analysis was carried out using SAS V.9.3 (SAS Institute, Cary, North Carolina, USA). Weighted Poisson regression taking into account the cluster sampling was used for univariate and multivariate analyses^25^ ^26^ to determine the association between condom use and the number of grey packets received in the past 3–12 months. Prevalence ratios (PRs) and adjusted PRs (APRs), with 95% CIs, were generated with the Poisson regression. Continuous variables were categorised. One model was created for each type of partner in the multivariable analysis. Variables from the univariate analysis were initially included in the multivariate model on the basis of a significance level of p<0.10. An iterative process was then used to keep only the variables that were found to be confounding in the model (eg, that changes the APR of the association between receiving grey packets and condom use by ≥10%). A test for trend was used to evaluate the dose–response relationship between the number of grey packets received and condom use. All p values shown are two-sided.

## Results

Sociodemographic, sex work-related and intervention exposure factors were measured among all 1378 FSWs with new or occasional clients, among whom 938 (68.7%) had had a repeat client. Tables 1 and 2 show the characteristics of the participants and univariate associations between each factor and CCU with the two types of partner.

**Table 1:** Sample characteristics and univariate associations between these characteristics and consistent condom use by FSWs with new or occasional clients in five districts of south India*†‡§

	N (%) (n=1378)	CCU (%)	Prevalence ratio (95% CI)	p Value
*Sociodemographic factors*				
District				0.024
Bangalore	369 (26.8)	63.7	0.97 (0.83 to 1.14)	
Bellary	198 (14.4)	70.2	1.11 (0.96 to 1.29)	
Guntur	208 (15.1)	79.8	1.20 (1.05 to 1.37)	
Belgaum	208 (15.1)	75.0	1.07 (0.92 to 1.23)	
Mumbai	395 (28.7)	68.6	Ref	
Age				0.175
<25	296 (21.5)	74.0	1.07 (0.97 to 1.18)	
25+	1082 (78.5)	69.1	Ref	
Marital status				0.004
Devadasi	156 (11.3)	79.5	1.18 (1.05 to 1.32)	
Never married	143 (10.4)	67.1	0.88 (0.73 to 1.06)	
Divorced/separated/widowed	651 (47.2)	68.8	1.00 (0.89 to 1.13)	
Currently married	428 (31.0)	69.9	Ref	
Literate				0.107
Yes	403 (29.2)	73.2	1.09 (0.98 to 1.21)	
No	975 (70.8)	68.9	Ref	
Age at first sex				0.562
<15	470 (34.1)	67.9	0.96 (0.82 to 1.11)	
15+	908 (65.9)	71.3	Ref	
*Sex work-related factors*				
Age at first sex work				0.004
<22	587 (42.6)	75.1	1.14 (1.04 to 1.24)	
22+	791 (57.4)	66.5	Ref	
Main place of solicitation				0.411
Brothel	315 (22.9)	68.3	1.06 (0.94 to 1.19)	
Home	318 (23.1)	74.8	1.09 (0.97 to 1.22)	
Rented room/lodge or other	82 (5.6)	72.0	1.10 (0.91 to 1.34)	
Public places	661 (48.0)	68.5	Ref	
Main place of entertaining				0.067
Brothel	349 (25.3)	78.2	1.17 (0.99 to 1.39)	
Home	394 (28.6)	66.8	1.11 (0.94 to 1.31)	
Rented room/lodge or other	523 (38.0)	68.5	1.02 (0.86 to 1.21)	
Public places	111 (8.1)	64.9	Ref	
Sex work sole income				0.187
Yes	476 (34.5)	70.3	1.06 (0.97 to 1.16)	
No	898 (65.2)	69.8	Ref	
FSW usually under influence of alcohol with partner				0.517
Yes	542 (39.3)	70.7	1.03 (0.94 to 1.14)	
No	834 (60.5)	70.0	Ref	
Partner usually under influence of alcohol				0.063
Yes	1189 (86.3)	75.8	0.86 (0.74 to 1.01)	
No	186 (13.5)	69.3	Ref	
Solicit independently				0.046
Yes	815 (59.1)	66.6	0.91 (0.83 to 1.00)	
No	560 (40.6)	75.4	Ref	
*Intervention exposure*				
Number of times contacted by staff in the past month				<0.001
<2	393 (28.5)	60.3	Ref	
2+	981 (71.2)	74.3	1.26 (1.10 to 1.44)	
Number of condom demos seen in the past month				0.002
0	257 (18.7)	57.2	Ref	
1	303 (22.0)	68.7	1.26 (1.02 to 1.56)	
2	363 (26.34)	76.6	1.44 (1.18 to 1.76)	
3+	397 (28.8)	76.8	1.36 (1.10 to 1.67)	
Test for trend				0.077
Duration since first contacted by intervention staff				0.001
Has not been contacted	229 (16.6)	54.1	Ref	
<1 (>0)	284 (20.6)	68.0	1.25 (1.01 to 1.54)	
1 year	284 (20.6)	72.9	1.35 (1.09 to 1.66)	
2 to 3 years	360 (26.1)	74.7	1.44 (1.18 to 1.74)	
4 years+	219 (15.9)	78.5	1.46 (1.19 to 1.79)	
Test for trend				0.039
Treatment of STIs: number of grey packets received in the past 3–12 months				0.259
0	668 (48.5)	68.7	Ref	
1	129 (9.4)	74.4	1.02 (0.88 to 1.18)	
2	254 (18.4)	74.4	1.10 (0.98 to 1.23)	
3+	326 (23.7)	68.1	0.88 (0.73 to 1.07)	
Test for trend				0.259

*Consistent condom use is defined as reporting always using condoms.

†Owing to missing values, the total N for each variable may be different from 1378 (total of FSW with a new or occasional client). The small number of missing values does not significantly affect the results.

‡Prevalence ratios are presented with a 95% CI.

§CCU is defined as consistent condom use.

FSW, female sex worker; STI, sexually transmitted infection.

**Table 2:** Sample characteristics and univariate associations between these characteristics and consistent condom use by FSWs with the most recent repeat client in five districts of south India*†‡§

	N (%) (n=1378)	CCU (%)	Prevalence ratio (95% CI)	p Value
*Sociodemographic factors*				
District				<0.001
Bangalore	221 (23.6)	64.7	1.13 (0.73 to 1.75)	
Bellary	95 (10.1)	67.3	1.56 (1.30 to 1.87)	
Guntur	181 (19.3)	85.6	1.74 (1.49 to 2.03)	
Belgaum	117 (12.5)	53.0	1.06 (0.83 to 1.36)	
Mumbai	324 (34.5)	51.5	Ref	
Age				0.045
<25	199 (21.2)	71.3	1.21 (1.00 to 1.46)	
25+	739 (78.8)	60.8	Ref	
Marital status				0.100
Devadasi	90 (9.5)	68.9	1.19 (0.99 to 1.42)	
Never married	115 (12.3)	66.1	0.94 (0.74 to 1.21)	
Divorced/separated/widowed	450 (48.0)	61.6	0.94 (0.73 to 1.20)	
Currently married	283 (30.1)	62.1	Ref	
Literate				0.958
Yes	307 (32.7)	64.5	1.01 (0.81 to 1.24)	
No	631 (67.3)	62.3	Ref	
Age at first sex				0.938
<15	311 (33.1)	61.4	0.99 (0.86 to 1.15)	
15+	627 (66.8)	63.8	Ref	
*Sex work-related factors*				
Age at first sex work				0.196
<22	418 (44.6)	66.8	1.15 (0.93 to 1.43)	
22+	520 (55.4)	60.0	Ref	
Main place of solicitation				0.084
Brothel	210 (22.4)	67.1	1.24 (0.98 to 1.57)	
Home	207 (22.1)	69.6	1.34 (1.06 to 1.68)	
Rented room/lodge or other	60 (6.4)	70.0	1.33 (0.99 to 1.79)	
Public places	459 (48.9)	57.1	Ref	
Main place of entertaining				0.192
Brothel	249 (26.5)	61.5	0.92 (0.76 to 1.11)	
Home	252 (26.9)	69.4	0.99 (0.81 to 1.20)	
Rented room/lodge or other	358 (38.2)	58.4	0.75 (0.56 to 1.01)	
Public places	78 (8.3)	68.0	Ref	
Sex work sole income				0.013
Yes	319 (34.0)	67.4	1.29 (1.06 to 1.56)	
No	616 (65.7)	60.7	Ref	
FSW usually under influence of alcohol with partner				0.007
Yes	378 (40.3)	55.0	0.79 (0.67 to 0.94)	
No	559 (59.6)	68.3	Ref	
Partner usually under influence of alcohol				0.047
Yes	603 (64.3)	58.0	0.85 (0.72 to 1.00)	
No	334 (35.6)	72.2	Ref	
Solicit independently				0.150
Yes	510 (54.4)	57.8	0.85 (0.68 to 1.06)	
No	427 (45.5)	69.1	Ref	
*Intervention exposure*				
Number of times contacted by staff in the past month				0.525
<2	238 (25.4)	57.1	Ref	
2+	697 (74.3)	65.3	1.08 (0.86 to 1.36)	
Number of condom demos seen in the past month				0.041
0	151 (16.1)	52.3	Ref	
1	198 (21.1)	62.1	1.09 (0.84 to 1.41)	
2	258 (27.5)	70.0	1.23 (0.97 to 1.57)	
3+	289 (30.8)	65.7	0.98 (0.65 to 1.47)	
Test for trend				0.222
Duration since first contacted by intervention staff				0.004
Has not been contacted	132 (14.1)	50.0	Ref	
<1 (>0)	180 (19.2)	65.0	0.94 (0.55 to 1.60)	
1 year	169 (18.0)	65.7	1.39 (0.98 to 1.71)	
2 to 3 years	270 (28.8)	68.9	1.43 (1.12 to 1.83)	
4 years+	185 (19.7)	58.9	1.09 (0.83 to 1.44)	
Test for trend				0.017
Treatment of STIs: number of grey packets received in the past 3–12 months				0.270
0	447 (47.7)	63.3	Ref	
1	80 (8.5)	71.3	1.08 (0.91 to 1.29)	
2	174 (18.6)	60.3	0.80 (0.56 to 1.13)	
3+	237 (25.3)	61.6	0.82 (0.64 to 1.05)	
Test for trend				0.270

*Consistent condom use is defined as reporting always using condoms.

†Owing to missing values, the total N for each variable may be different from 938 (total of FSW with a most recent repeat client). The small number of missing values does not significantly affect the results.

‡Prevalence ratios are presented with a 95% CI.

§CCU is defined as consistent condom use.

FSW, female sex worker; STI, sexually transmitted infection.

The majority of FSWs (78.5%) were older than 25 years and were illiterate (70.8%). Seventy per cent reported using condoms consistently with their new or occasional clients, whereas 591 (63.0%) of FSWs, who reported repeat clients, reported CCU with their most recent one. Less than half (48.5%) of the FSWs had not received a grey packet in the past 3–12 months, while 129 (9.4%) had received one treatment, 254 (18.4%) had received two treatments and 326 (23.7%) had received three treatments or more.

In univariate analysis (tables 1 and 2), CCU was higher among FSWs who had seen condom demonstrations in the past month in comparison to those who had not seen any. CCU was at its highest level for two condom demonstrations seen in the past month for both FSWs with new or occasional clients (PR 1.44, p<0.001) as well as for FSWs with a most recent repeat client (PR 1.23, p=0.095). CCU was also higher among FSWs who had been contacted by intervention staff in comparison to those who had not been contacted. CCU increased when time since first contacted by intervention staff increased and was at its highest level at 4 years and more for new and occasional clients (PR 1.46, p<0.001); it was as its at its highest level at 2 or 3 years since first contacted for the most recent repeat client (PR 1.43, p=0.004). CCU with new and occasional clients was higher among FSWs who had two or more contacts with intervention staff in the past month for PR 1.26 (p<0.001), but results were not significant for FSWs with the most recent repeat client. In univariate analysis, no significant association was found between CCU and the number of grey packets received in the past 3–12 months. For the sociodemographic factors, district was associated with CCU with both types of partner (new/occasional clients p=0.024 and the most recent repeat client p<0.001). Age was only significant for FSWs with the most recent repeat client (PR 1.21, p=0.045). For sex work-related factors, alcohol intake by the FSW with clients was only significantly associated with CCU with the most recent repeat client (PR 0.79, p=0.007), but was significant for both types in the case for alcohol intake by the client (new/occasional clients: PR 0.85, p=0.063 and the most recent repeat client: PR 0.85, p=0.047).

In multivariate analysis (table 3), final models were adjusted for the district, the number of condom demonstrations seen in the past month and the time since first contacted by intervention staff.

**Table 3:** Multivariate associations between these characteristics and CCU by FSWs with new or occasional clients and with a most recent repeat client in five districts of south India*†‡

	CCU with new or occasional clients		CCU with most recent repeat client	
	Adjusted prevalence ratio (95% CI)	p Value	Adjusted prevalence ratio (95% CI)	p Value
Number of grey packets received in the past 3–12 months (vs 0)				
1	0.92 (0.80 to 1.06)	0.258	0.90 (0.75 to 1.09)	0.276
2	0.90 (0.79 to 1.02)	0.089	0.63 (0.47 to 0.85)	0.003
3	0.70 (0.57 to 0.84)	<0.001	0.63 (0.51 to 0.78)	<0.001
Test for trend		<0.001		<0.001
District				
Bangalore	1.14 (0.95 to 1.36)	0.165	1.51 (1.17 to 1.95)	0.002
Bellary	1.23 (1.04 to 1.45)	0.015	1.93 (1.48 to 2.52)	<0.001
Guntur	1.32 (1.14 to 1.53)	<0.001	1.85 (1.58 to 2.18)	<0.001
Belgaum	1.27 (1.08 to 1.50)	0.004	1.24 (0.93 to 1.64)	0.142
Mumbai	Ref		Ref	
Number of condom demos seen in the past month (vs 0)				
1	1.18 (0.69 to 2.02)	0.545	0.88 (0.58 to 1.32)	0.531
2	1.34 (0.79 to 2.29)	0.278	0.94 (0.63 to 1.40)	0.750
3+	1.36 (0.80 to 2.32)	0.253	0.87 (0.56 to 1.36)	0.549
Test for trend		0.025		0.834
Duration since first contacted by intervention staff (vs never contacted)				
<1 year	1.03 (0.59 to 1.80)	0.911	1.13 (0.65 to 1.97)	0.664
1	1.15 (0.64 to 2.05)	0.646	1.70 (1.07 to 2.70)	0.024
2	1.21 (0.69 to 2.12)	0.502	1.96 (1.22 to 3.14)	0.006
3+	1.32 (0.76 to 2.27)	0.325	1.62 (1.04 to 2.53)	0.034
Test for trend		<0.001		0.033

*CCU is defined as reporting always using condoms.

†Models were adjusted for variables that were significantly associated with the main outcome, CCU, on a p<0.10 significance level in univariate analysis and were found to be confounders of the association between CCU and PPT. For CCU with new or occasional clients and for the most recent repeat client, final models were adjusted for district, the number of condom demos seen in the past month and the duration since first contacted by intervention staff.

‡Adjusted prevalence ratios are presented with a 95% CI.

CCU, consistent condom use; FSW, female sex worker; PPT, periodic presumptive treatment.

Tests for trend were carried out to evaluate the dose relationship between CCU and continuous variables. For the variable number of grey packets received in the past 3–12 months, tests for trends were significant for both new or occasional clients (p<0.001) and the most recent repeat client (p<0.001). Results show that CCU fell when the number of grey packets received increased. The lowest CCU was observed when FSWs received three grey packets or more, both with new or occasional clients (APR 0.70, 95% CI 0.57 to 0.84, p<0.001) and the most recent repeat client (APR 0.63, 95% CI 0.51 to 0.78, p<0.001). No significant association was found between receiving one treatment and CCU with new or occasional clients (APR 0.92, 95% CI 0.80 to 1.06, p=0.258) and with the most recent repeat client (APR 0.90, 95% CI 0.75 to 1.09, p=0.276).

## Discussion

Results from this study show an association between the use of grey packets and lower levels of CCU among FSWs in the context of the *Avahan* intervention in India. After adjusting for confounding factors, a dose–response relationship was found between the number of grey packets received and CCU with new and occasional clients, and with the most recent repeat client. However, the results were only significant when FSWs had received two or more grey packets in the past 3–12 months.

There are several explanations possible for this association. Owing to the cross-sectional nature of the study and the way the questions were asked, we could not make sure if condom use was already inconsistent before the FSW took the treatment or if it became inconsistent after taking the treatment.

On the one hand, if condom use was already inconsistent, the explanation for the association could be twofold: first, women who did not use condoms consistently attended the clinic more often than others as a compensatory action and were thus more likely to receive grey packets for PPT; second, FSWs with a higher burden of cervicitis due to possible inconsistent condom use were more likely to present with symptoms of vaginal discharge and receive grey packets in the context of syndromic management.

On the other hand, given the relatively low prevalence of gonorrhoea and chlamydia (3.5% and 6.5%, respectively) among FSWs in prevalence studies conducted in the context of *Avahan*,^27^ it is most likely that the vast majority of cases of vaginal discharge consulting at the clinics did not have cervicitis but rather vaginitis, a condition less clearly related to inconsistent condom use. In addition, the way the question was asked about condom use refers to recent use, whereas the question on grey packets covered a 3-month to 12-month period. It is thus likely that in many instances, inconsistent condom use came after treatment. This plausible hypothesis thus suggests that a possible disinhibiting effect of receiving an STI treatment would lead to more unsafe sex, in the context where appropriate information is not always conveyed to the patients. Furthermore, the importance of good communication between physicians and patients has not been extensively studied in this type of context. However, good communication has been shown to have an important impact on various patient health outcomes, adherence to treatment and patient satisfaction in other settings.^28–31^ In our study, it is possible that the lack of adequate information and poor communication about the treatment led to a false feeling of security and disinhibition, which resulted in reduced condom use. Thus, there is a need to improve communication about the treatment and about the need for continued and CCU. Such a false feeling of security was revealed in a qualitative study carried out in Guntur district, in conjunction with the quantitative study used in the present analysis. Here is what some of the interviewed FSWs said:^15^

It is said that sex workers get AIDS. It appears as sores and itching develops near the vagina when one has AIDS. It cannot be cured with medicines. The organization gives medicines to prevent such things from happening.

To have sex without a condom and to avoid AIDS, one should wash thoroughly with hot water and use good medicines.

On the other hand, almost one-third of the study population in our study were illiterate (29.2%) and a lot of them had wrong beliefs about HIV and treatments of STIs in general, as also noted in the citations above. This could have contributed to a wrong understanding of the effect of the treatment, even if the physicians were giving clear explanations.

Furthermore, risk compensation can be observed with those types of interventions.^32^ It has been observed in other contexts, for example, in male circumcision interventions.^33^ ^34^ In a study taking place in rural areas of South Africa, condom and STI knowledge was found to have an impact on condom use. Since the community had wrong beliefs about circumcision and its protective effect, lower condom use was observed among circumcised men.^33^ A rise in HIV incidence among MSM was also observed in many developed countries, as a consequence of an increase in condomless sex, following the availability of highly active antiretroviral therapy.^35^ Few studies were available regarding disinhibition after PPT. However, the few studies that have examined trends in condom use following implementation of PPT programmes did not report any evidence of increased risk taking.^6^ ^8^

Unfortunately, it was impossible to determine if the treatment was given presumptively or not with our data. This is the main limitation of the present study. Consequently, further studies would be needed to examine if PPT could lead to inconsistent condom use. This study also has other limitations. All data were self-reported and could therefore be susceptible to a social desirability bias since many questions were socially sensitive. In addition, many FSWs are mobile and difficult to reach and so this study may not represent all types of FSWs.

The condom use levels in our study were moderately high, which is in line with the results of the first round of the integrated biological and behavioural assessment (IBBA) surveys that were conducted in the same period.^16^ CCU with clients in general was 65.2% in IBBA round 1. Several years after the implementation of the *Avahan* programme, in IBBA round 2 conducted in 2009, CCU reached 84.0%.^36^ In general terms, the strength of the *Avahan* programme was mostly in the behavioural and community components, as regular coverage of FSWs by these components of the intervention was a lot higher than regular clinical coverage.^37^ Nevertheless, most reports suggest that *Avahan* had a major impact on the HIV epidemic among both FSWs^27^ ^36^ ^38^ and the general population^39^ in the states of south India covered by the intervention.

In conclusion, the results of our study suggest an association between receiving an STI treatment and inconsistent condom use in the early days of the *Avahan* programme in India. Although the directionality of the association cannot be fully determined with our data, this observation underlines the importance of both improving counselling about the effect of STI treatment in clinical settings and implementing a broad range of strategies for HIV prevention among FSWs. Such strategies, implemented within a combination framework, could notably include regular HIV testing followed by pre-exposure prophylaxis for HIV-negative FSWs and immediate treatment (often called treatment as prevention) for those found infected.^40^ Finally, further studies are needed to clarify the relationship between the use of STI treatments and condom use, especially regarding the effect of PPT on condom use.
